# Contribution of *MLH1* constitutional methylation for Lynch syndrome diagnosis in patients with tumor MLH1 downregulation

**DOI:** 10.1002/cam4.1285

**Published:** 2018-01-17

**Authors:** Diana Pinto, Carla Pinto, Joana Guerra, Manuela Pinheiro, Rui Santos, Hege Marie Vedeld, Zeremariam Yohannes, Ana Peixoto, Catarina Santos, Pedro Pinto, Paula Lopes, Ragnhild Lothe, Guro Elisabeth Lind, Rui Henrique, Manuel R. Teixeira

**Affiliations:** ^1^ Cancer Genetics Group IPO Porto Research Center (CI‐IPOP) Portuguese Oncology Institute of Porto (IPO Porto) Porto Portugal; ^2^ Department of Genetics Portuguese Oncology Institute of Porto (IPO Porto) Porto Portugal; ^3^ Department of Molecular Oncology Institute for Cancer Research Oslo University Hospital Norway; ^4^ Department of Pathology Portuguese Oncology Institute of Porto (IPO Porto) Porto Portugal; ^5^ Cancer Biology and Epigenetics Group IPO Porto Research Center (CI‐IPOP) Portuguese Oncology Institute of Porto (IPO Porto) Porto Portugal; ^6^ Institute of Biomedical Sciences Abel Salazar (ICBAS) University of Porto Porto Portugal

**Keywords:** Cancer predisposition, Lynch syndrome, *MLH1* constitutional methylation, MS‐MPLA

## Abstract

Constitutional epimutation of the two major mismatch repair genes, *MLH1* and *MSH2*, has been identified as an alternative mechanism that predisposes to the development of Lynch syndrome. In the present work, we aimed to investigate the prevalence of *MLH1* constitutional methylation in colorectal cancer (CRC) patients with abnormal expression of the MLH1 protein in their tumors. In a series of 38 patients who met clinical criteria for Lynch syndrome genetic testing, with loss of MLH1 expression in the tumor and with no germline mutations in the *MLH1* gene (35/38) or with tumors presenting the *BRAF* p.Val600Glu mutation (3/38), we screened for constitutional methylation of the *MLH1* gene promoter using methylation‐specific multiplex ligation‐dependent probe amplification (MS‐MLPA) in various biological samples. We found four (4/38; 10.5%) patients with constitutional methylation in the *MLH1* gene promoter. RNA studies demonstrated decreased *MLH1* expression in the cases with constitutional methylation when compared with controls. We could infer the mosaic nature of *MLH1* constitutional hypermethylation in tissues originated from different embryonic germ layers, and in one family we could show that it occurred de novo. We conclude that constitutional *MLH1* methylation occurs in a significant proportion of patients who have loss of MLH1 protein expression in their tumors and no *MLH1* pathogenic germline mutation. Furthermore, we provide evidence that *MLH1* constitutional hypermethylation is the molecular mechanism behind about 3% of Lynch syndrome families diagnosed in our institution, especially in patients with early onset or multiple primary tumors without significant family history.

## Introduction

Lynch syndrome is the most common hereditary syndrome that predisposes to colorectal cancer (CRC), corresponding to 2‐5% of all CRCs 1, 2. It is an autosomal dominant disease caused by germline mutations in the Mismatch Repair (MMR) genes involved in DNA repair 3, 4. These include *MLH1*,* MSH2*,* MSH6,* and *PMS2*, although about 90% of the mutations described in this syndrome occur in *MLH1* or *MSH2* 5, 6, 7, 8. In the past decade, another distinct mechanism affecting the two key MMR genes *MLH1* and *MSH2* was unraveled in a subset of patients meeting the clinical criteria for LS without a germline MMR mutation, termed as “constitutional epimutations” or just “epimutation” 9, 10. This phenomenon consists in constitutional transcriptional silencing of these genes by epigenetic mechanisms rather than by genetic mutations that directly affect the sequence of the gene 8, 9, 10.

Constitutional epimutations are stable epigenetic abnormalities that are present in normal tissues 11. Constitutional epimutations of the *MSH2* gene are secondary to germline deletions in the *EPCAM* gene in *cis*, being transmitted in an autosomal dominant fashion just like germline MMR mutations 12. However, constitutional epimutations of the *MLH1* gene are more variable, and the pattern of transmission of these distinct forms of *MLH1* epimutation presumably reflects their mechanistic basis. *MLH1* epimutations may be dichotomized into two categories: (1) those that arise spontaneously and are reversible between generations, though occasionally transmitted to the next generation in a non‐Mendelian pattern (primary *MLH1* epimutation); and (2) Mendelian epimutations that follow a classic autosomal dominant inheritance pattern due to an underlying *cis*‐genetic cause (secondary/genetically facilitated *MLH1* epimutation) 10, 13.

Few cases of constitutional *MLH1* methylation have so far been reported 10, 11, 12, 13. Furthermore, the exact prevalence of *MLH1* constitutional epimutations is still unknown, as most studies addressing this issue were based on series enriched for patients with CRC diagnosed at an age of onset below 50 years 8, 14, 15, 16.

## Materials and Methods

### Patients, samples, and DNA extraction

The study includes a consecutive series of peripheral blood lymphocyte (PBL) samples from 38 patients (index cases, 17 males and 21 females), who meet clinical criteria for Lynch syndrome genetic testing and had loss of MLH1 expression in the tumor (relevant clinical information in Table 1). Three of these patients (3/38) presented the p.Val600Glu *BRAF* somatic mutation (and therefore were presumed not to carry a germline mutation), whereas the remaining 35 had a negative genetic test for *MLH1* deleterious germline mutations. These patients were diagnosed and surgically treated at the Portuguese Oncology Institute of Porto, assessed through genetic counseling, and referred to the Genetics Department of this institution between 2009 and 2014. The majority of these patients (37/38; 97.4%) met the Bethesda criteria and one family (1/38; 2.6%) met the Amsterdam criteria. Clinicopathological information was obtained from medical records.

**Table 1:** Clinicopathological data of 38 index cases fulfilling the clinical criteria for Lynch syndrome

Patient	Gender	Tumor localization (diagnosis age)	IHC MMR	Clinical criteria	*BRAF*	*MLH1*
#1	F	Ascending colon (40)	MLH1/PMS2 absence	BC	NA	WT
#2	F	Stomach (75)Cecum^b^ (75)Breast (78)	MLH1 absence^a^	BC	NA	WT
#3	M	Descending colon (38 and 48)	MLH1 absence^a^	BC	WT	WT
#4	M	Ascending colon (25)	MLH1/PMS2 absence	BC	WT	WT
#5	M	Sigmoid colon (51)	MLH1/PMS2 absence	BC	WT	WT
#6	M	Rectum (53)	MLH1/PMS2 with decreased immunoreactivity	BC	WT	WT
#7	M	Ascending colon (43)	MLH1/PMS2 absence	BC	WT	WT
#8	F	Rectum (16)	MLH1 absence (normal PMS2)	BC	WT	WT
#9	F	Rectum (43)	MLH1 absence^a^	BC	V600E	NA
#10	F	Ascending colon^b^ (26)Stomach (60)	MLH1/PMS2 absence	BC	WT	WT
#11	M	Ascending colon (65)	MLH1/PMS2 absence	BC	WT	WT
#12	F	Ascending colon (62)	MLH1/PMS2 absence	BC	WT	WT
#13	M	Sigmoid colon (44)	MLH1/PMS2 absence	BC	WT	WT
#14	F	Ascending colon (69)	MLH1/PMS2 absence	BC	WT	WT
#15	F	Uterus and ovary (38)	MLH1/PMS2 absence	BC	WT	WT
#16	F	Breast (60)Ascending colon^b^ (66)	MLH1/PMS2 absence	BC	WT	WT
#17	M	Ascending colon (25)	MLH1/PMS2 absence	BC	WT	WT
#18	M	Sigmoid colon (43)	MLH1/PMS2 absence	BC	NA	WT
#19	M	Sigmoid colon (47)	MLH1/PMS2 absence	BC	WT	WT
#20	M	Ascending colon (23)	MLH1/PMS2 absence	BC	WT	WT
#21	F	Endometrium (57)Ascending Colon^b^ (74)Lung (74)	MLH1/PMS2 absence	BC	V600E	NA
#22	F	Sigmoid colon (47)	MLH1/PMS2 absence	BC	WT	WT
#23	F	Ascending colon (59)	MLH1/PMS2 absence	BC	WT	WT
#24	M	Rectum (45)Ascending colon^b^ (61)	MLH1/PMS2 absence	BC	WT	WT
#25	F	Ascending colon (62)Endometrium^b^ (63)	MLH1/PMS2 absence	BC	WT	WT
#26	F	Ascending colon (41)	MLH1/PMS2 absence	BC	WT	WT
#27	M	Rectum (33)	MLH1/PMS2 absence	BC	WT	WT
#28	F	Stomach (78)	MLH1/PMS2 absence	BC	WT	WT
#29	F	Breast (30)	MLH1/PMS2 absence^a^	AC	WT	WT
#30	M	Sigmoid colon (61)	MLH1/PMS2 absence	BC	WT	WT
#31	F	Descending colon (65)	MLH1/PMS2 absence	BC	V600E	NA
#32	F	Ascending colon (54)	MLH1/PMS2 absence	BC	WT	WT
#33	F	Ascending colon (42)	MLH1/PMS2 absence	BC	NA	WT
#34	M	Ascending colon (60)	MLH1/PMS2 absence	BC	WT	WT
#35	M	Ascending colon (44)	MLH1/PMS2 absence	BC	NA	WT
#36	F	Endometrium (50)	MLH1/PMS2 absence	BC	WT	WT
#37	F	Sigmoid colon (56)	MLH1/PMS2 with decreased immunoreactivity	BC	WT	WT
#38	M	Ascending colon^b^ (48)Transverse colon (48)Descending colon (48)	MLH1/PMS2 absence	BC	WT	WT

AC, Amsterdam criteria; BC, Bethesda criteria; F, female; IHC, immunohistochemistry; M, male; MMR, mismatch repair; NA, not analyzed; WT, wild‐type.

The analysis was not performed for PMS2.

a IHC was performed on tumor of a relative.

b Tumor used for IHC MMR (when cases presented multiple tumors).

Whenever possible, family members of the index patients were also studied, along with buccal swab samples and paraffin‐embedded tissue samples (with different germ layer origins) from patients harboring constitutional epimutation. DNA from PBL samples was obtained using the Magna Pure LC 2.0 (Roche Applied Science, Indianapolis, Indiana) and RNA extraction from PBL was performed using Trizol reagent (Invitrogen) according to the manufacturer's instructions and standard protocol 17. The DNA in paraffin histological sections was isolated with the QIAamp^®^ DNA FFPE Tissue Kit (Qiagen, Hilden, Germany) following the manufacturer's instructions. The buccal mucosa was collected with a swab and preserved in a dry environment and DNA was extracted according to standard procedures.

### Methylation analysis by MS‐MLPA

Analysis of *MLH1* promoter methylation was performed on PBL DNA of the index patients. Methylation testing was performed by Methylation‐Specific Multiplex Ligation‐Dependent Probe Amplification (MS‐MLPA) using the SALSA MS‐MLPA ME011‐B1 kit (MRC Holland, Amsterdam, Netherlands). Whenever possible, buccal swab samples and paraffin‐embedded tissues were analyzed for *MLH1* promoter methylation using the same approach. The six probe pairs in the *MLH1* promoter (with the respective ligation sites located at −659, −518, −382, −246, −13, and +206 relative to the start codon, LRG_216t1) cover independent regions: regions A to D of the promoter and intron 1. The most important methylation region associated with *MLH1* silencing is the C‐Deng region, from −248 to −178nt before the transcription site, and the second most important region is the D‐Deng region, from −9 to +15 nt 14, 18. Fragments were analyzed using the GeneMapper software (Applied Biosystems, Foster City, CA, USA). DNA Quantity (Q‐fragments) and DNA Denaturation (D‐fragments) control fragments present in the MS‐MLPA probe mix were checked. Aberrant methylation was assessed by comparison with normal reference samples. Two intraexperimental replicates in two independent experiments were analyzed and the mean was calculated. In all cases with *MLH1* promoter constitutional methylation an independent DNA PBL extraction was performed and subsequently analyzed by MS‐MLPA.

Additionally, the SALSA MLPA probemix ME042‐C1 CIMP kit (MRC Holland) was used as described above in PBL samples. This probemix enables detection of aberrant methylation of CpG islands around the transcription start site of eight genes (*CACNA1G, CDKN2A, CRABP1, IGF2, MLH1, NEUROG1, RUNX3,* and *SOCS1*), for which an altered methylation status, related to the CpG Island Methylator Phenotype (CIMP), has been reported in CRC 19, 20.

### Bisulfite treatment and DNA methylation analyses by qMSP and ddPCR

DNA from PBL samples (patient #3, #10, #27, #38, and parents of patient #27) was available for bisulfite treatment. The EpiTect Bisulfite kit (Qiagen) was used for the conversion, following the manufacturer’s protocol. The samples were purified using the QIAcube (Qiagen).

Quantitative methylation‐specific PCR (qMSP) was used to analyze the C‐region of the *MLH1* promoter (assay location: −270→−194 relative to the transcription start site; forward primer: GCGGATAGCGATTTTTAACGC, reverse primer: CTTCGTCCCTCCCTAAAACGA, probe: 6FAM‐AGCGTATATTTTTTTAGGTAGCG‐MGB) 21. In addition, a panel of six biomarkers (previously shown to be hypermethylated in 65–94% of tumor samples from colorectal cancer patients 22) comprising *CNRIP1*,* FBN1*,* INA*,* MAL*,* SNCA*, and *SPG20* was analyzed, using the same primer and probe sequences as previously described 22, 23. The ALU‐C4 element 24 was used as a control to normalize for DNA input. A fivefold dilution standard curve (32.5–0.052 ng) was generated from in vitro methylated DNA (IVD; Zymo Research, Irvine, CA) and added in triplicate to 384 well plates together with 3 × 32.5 ng bisulfite‐treated DNA template, 3× water (negative control), 3 × 32.5 ng bisulfite‐treated DNA isolated from the whole blood of healthy individuals (methylation negative control), and two 3 × 32.5 ng bisulfite converted IVD (methylation‐positive controls). 1 x TaqMan Universal PCR Mastermix No AmpErase UNG (Life Technologies), 0.9 *μ*mol/L of each primer, and 0.2 *μ*mol/L probe was added to each well to a total reaction volume of 20 *μ*L. The PCR reactions (95°C for 10 min, followed by 40 cycles of 95°C for 15 sec and 60°C for 60 sec) were carried out in a 7900HT Real‐Time PCR System (Life Technologies, Carlsbad, CA).

Samples amplified after cycle 35 were censored according to the recommendations from Life Technologies. To normalize for DNA input, the median quantity of the GENE was divided by the median quantity of ALU 24. The percent of methylated reference (PMR) values were calculated by dividing the normalized quantity of the GENE by the averaged normalized quantity of the two positive control controls, and multiply by 100.

Droplet digital PCR (ddPCR) of the *MLH1* promoter was performed on bisulfite‐treated PBL samples from the parents of patient #27, using the same *MLH1* assay as for qMSP, with a FAM‐labeled probe. In addition, a 4plex control assay, marked with VIC, was included to ensure that enough DNA was present for amplification. The ddPCR reactions were performed using the QX200^™^ droplet digital PCR platform (BioRad, Hercules, CA) following the manufactures’ protocol. Droplets were generated by the QX200 Droplet Generator. Following PCR amplification, the fluorescence signals were measured by the QX200 Droplet Reader and analyzed by QuantaSoft version 1.7.4.0917 (BioRad).

### *MLH1* promoter sequencing

Screening for mutations within the *MLH1* promoter (which may affect the binding of MLPA probes) was performed by Sanger sequencing in the 38 peripheral blood samples and using two sets of primers, according to Pineda et al. 25.

### *MLH1* transcript quantification in PBL samples

The *MLH1* transcripts were quantified by semiquantitative multiplex RT‐PCR. For this purpose, we simultaneously amplified a control transcript, the *β*2‐microglobulin (*B2M*), and part of the *MLH1* transcript, using fluorescence‐labeled primers and according to the QIAGEN OneStep RT‐PCR Kit (Qiagen). *MLH1* transcript levels were calculated by comparing the relative peak areas of the *MLH1* transcript to the relative peak areas of the *B2M*. Three independent experiments were performed.

### *MLH1* allelic expression analysis in PBL samples

In all patients with constitutional methylation and heterozygous for the coding *MLH1* polymorphism c.655A>G (rs1799977) in exon 8, allelic expression analyses were determined in genomic DNA and in cDNA by single‐nucleotide primer extension (SNuPE) using the SNaPshot Kit (Applied Biosystems) following the manufacturer′s protocol. The results were independently scored by two observers, and a third round of analyses confirmed the results.

### Analysis of p.Val600Glu *BRAF* mutation

The *BRAF* c.1799T>A, p.Val600Glu (also known as V600E), mutation was screened in the tumors of all 38 patients by PCR amplification and High Resolution Melting (HRM) analysis on a LightCycler‐480 II Real‐Time System (Roche Applied Science, Indianapolis, Indiana). As a confirmation of this technique SNuPE was performed following the SNaPshot Kit (Applied Biosystems) manufacturer's protocol.

### Study of microsatellite instability

Microsatellite instability was performed using the Bethesda panel (BAT25, BAT26, D2S123, D5S346, and D17S250), according to the 1997 National Cancer Institute Guidelines using fluorescence‐labeled primers 26. Fragments were analyzed for length variations on an ABI PRISM^™^ 310 Genetic Analyzer DNA sequencer (Applied Biosystems) and allele sizes were determined using the GeneMapper software. The results were independently scored by two observers and an additional round of analyses confirmed the results.

## Results

### Identification of patients harboring constitutional *MLH1* promoter methylation

**Figure 1:**
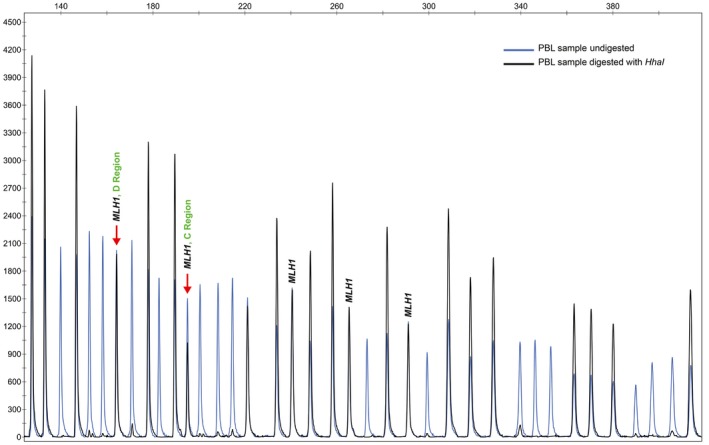
GeneMapper MS‐MLPA electropherogram plot of one replicate of patient #27 PBL DNA presenting constitutional *MLH1* methylation. The C‐ and D‐Deng regions, the most important regions associated with the transcription of *MLH1* gene, are highlighted with the red arrows.

Constitutional methylation of the *MLH1* promoter was detected in four (4/38; 10.5%) patients (Table 2 and 3; Table S1; Fig. 1), namely cases #3, #10, #27, and #38. The mean age at diagnosis of these patients was 36 years (range 26–48). The methylation level detected in PBL at the C‐Deng region (the region most directly involved in *MLH1* transcriptional activity) by MS‐MLPA was 14.1%, 18.3%, 32.6%, and 46.3% for patient #3, #10, #27, and #38, respectively, indicating that this alteration, at least for some of these patients, might be present in mosaic. *MLH1* promoter methylation was also detected by qMSP analyses in the PBL samples from the four probands (Table 3).

**Table 2:** *MLH1* methylation levels (%) using MS‐MLPA in samples from different germline origins in the four probands with constitutional epimutations

	% *MLH1* methylation									
	PBL		Tumor		Normal colon mucosa		Buccal mucosa		Muscle	
Patient	C region (‐246 nt)	D region (−13 nt)	C region (−246 nt)	D region (−13 nt)	C region (−246 nt)	D region (−13 nt)	C region (−246 nt)	D region (−13 nt)	C region (−246 nt)	D region (−13 nt)
#3	14.1	43.3	20.4	62.9	8.35	27.7	12.4	44.5	11.1	32.3
#10	18.3	49.9	11	32.2	9.1	30.5	NA^a^	NA^a^	NA^a^	NA^a^
#27	32.6	51.4	13	38.6	14.4	35.4	13.8	40.5	12.2	39.5
#38	46.3	52.3	16.4	40.2	11.5	36	11.6	36.4	NA	NA

NA, not available; PBL, peripheral blood lymphocytes.

a Patient died during the study, so it was not possible to study buccal mucosa.

**Table 3:** Results of constitutional methylation and expression analyses of *MLH1*

	% Methylation in *MLH1* C‐region (gDNA)		% *MLH1* transcript levels decrease (cDNA)	% decrease A/G allele (cDNA/gDNA)
Patients	MS‐MLPA	qMSP	RT‐PCR	SNuPe
#3	14.1	35.4	38.0	NA
#10	18.3	37.0	37.4	52.5
#27	32.6	38.2	46.2	31.6
#38	46.3	41.9	40.7	NA

Additionally to PBL samples (mesoderm), we also studied *MLH1* promoter methylation in samples representative of all embryonic layers, namely: tumor and normal colon mucosa (endoderm), buccal mucosa (ectoderm), and muscle (mesoderm). *MLH1* methylation was present in all tissues analyzed (Table 2), demonstrating that this epigenetic alteration affects tissues from different embryonic origins. On the other hand, analysis of the constitutional methylation status using the MS‐MLPA CIMP kit in the four patients with *MLH1* epimutation showed that none of the other seven genes tested (*CACNA1G*,* CDKN2A*,* CRABP1*,* IGF2*,* NEUROG1*,* RUNX3,* and *SOCS1*) presented hypermethylation (Table S2). Additionally, the promoter regions of a panel frequently methylated in CRC 22 (*CNRIP1*,* FBN1*,* INA*,* MAL*,* SNCA*, and *SPG20*) were also unmethylated in the PBL samples from all four probands (Table S3).

We were able to study three relatives (parents and sister) of one of the probands with constitutional methylation of the *MLH1* promoter (patient #27), and none of them presented constitutional methylation of the *MLH1* promoter by MS‐MLPA. The absence of constitutional methylation in the parents was confirmed by ddPCR.

### Clinicopathological features of patients with *MLH1* constitutional methylation

**Figure 2:**
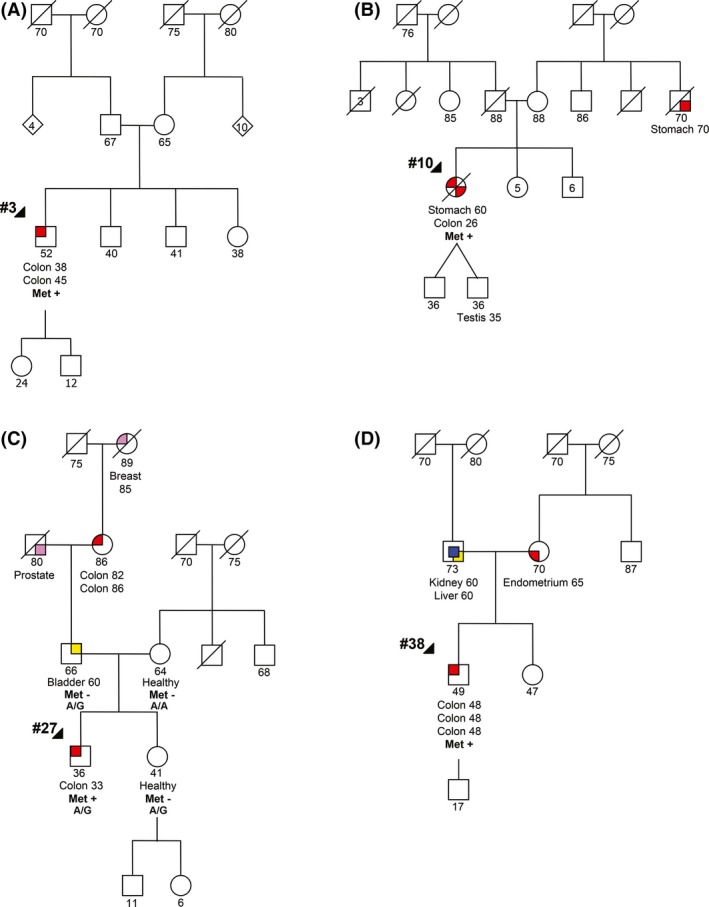
Family pedigrees of four patients positive for constitutional methylation of the *MLH1* promoter: (A) #3; (B) #10; (C) #27; (D) #38). Black arrows indicate the probands. A/A and A/G homozygous and heterozygous for the rs1799977, respectively; met‐, with no methylation of the *MLH1* promoter; met+, with methylation of the *MLH1* promoter. Circles, females; squares, males; semifilled, cancer affected.

Case #3 was a male who had two tumors of the descending colon, one at 38 and another at 45 years of age (primary metachronous tumors). The patient had no family history of cancer as shown in the family pedigree (Fig. 2A).

Case #10 was a female who was diagnosed with a moderately differentiated adenocarcinoma in the ascending colon at the age of 26 years and a stomach adenocarcinoma at the age of 60 years, the latter being her cause of death. This patient presented scant family history of cancer, namely a maternal uncle affected with gastric cancer at the age of 70 years (Fig. 2B).

Case #27 was a male who was diagnosed with an invasive adenocarcinoma in the rectum at the age of 33 years. This patient has family history of cancer, namely, a paternal great‐grandmother with breast cancer, a paternal grandfather with prostate cancer, a paternal grandmother with colon cancer, and a father with bladder cancer. The father and the healthy mother and sister also participated in this study (Fig. 2C).

Case #38 was a male who was diagnosed with three synchronous adenocarcinomas in the ascending, transverse, and descending colon at age of 48 years. The patient′s father was affected by kidney and liver cancer at the age of 60 years and the mother was affected with an endometrial cancer at the age of 65 years (Fig. 2D).

### Microsatellite instability and *BRAF* analyses

Microsatellite instability analysis was performed by fragment analysis in tumors and normal colon mucosa of all four patients positive for constitutional methylation. The tumors of all cases with constitutional methylation of the *MLH1* promoter were MSI‐H, with cases #3, #10, and #27 showing instability in 50% of the markers and case #38 in 100% of the markers (data not shown). On the other hand, none of the tumors of the four patients with constitutional methylation of the *MLH1* promoter presented the p.Val600Glu *BRAF* mutation.

### *MLH1* promoter sequencing

Sanger sequencing of the whole *MLH1* promoter (from c.−1469 to intron 1) was performed in PBL samples from all cases, probands (*n* = 38) and relatives (*n* = 3), in order to find out if there were any variants which might inhibit the binding of the MS‐MLPA probes and to identify promoter variants that might be associated with *MLH1* methylation. The results showed that 36 cases did not present variants affecting the binding sites of the MS‐MLPA probes or the *HhaI* restriction sites. Nonetheless, one patient, who did not present constitutional methylation*,* had the alteration *MLH1* c.‐261G>A (rs587782685) described as a variant of unknown significance (VUS) in the ClinVar NCBI database (http://www.ncbi.nih.gov/clinvar). Another heterozygous alteration, the SNP *MLH1* c.‐269C>G (rs35032294), was found in patient #38. Although these variants occur within a MS‐MLPA probe, the fact that no copy number changes were found indicates that they most probably did not affect the hybridization. Finally, the common SNP *MLH1* c.‐93G>A (rs1800734) was found in heterozygosity in 16 cases (16/38; 42%), including patient #3 with constitutional methylation, and in homozygosity in one case (1/38; 2.6%). This common polymorphism is outside the probe hybridization sites. No other variants were found in the *MLH1* promoter region.

### Quantification of the *MLH1* transcript

**Figure 3:**
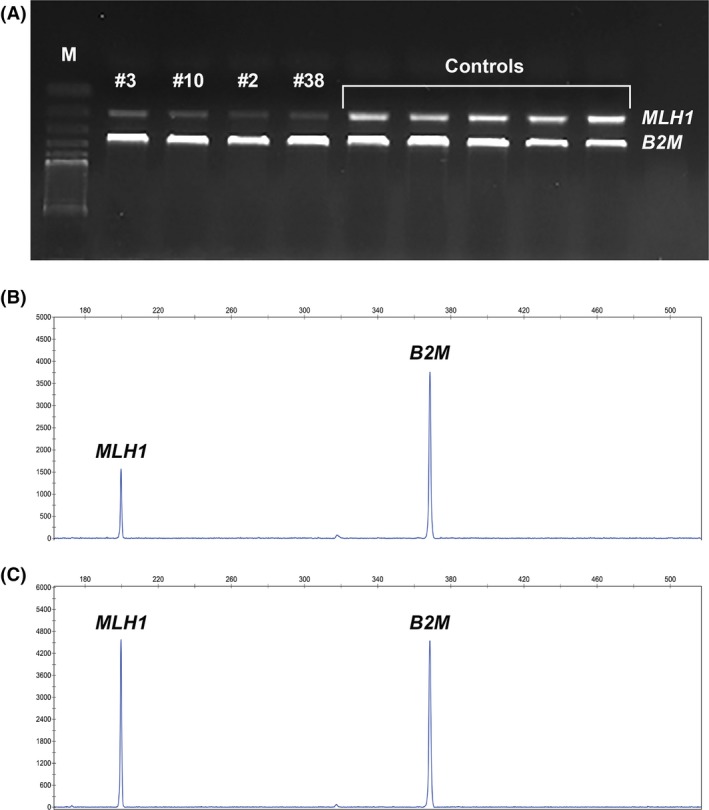
(A) Electrophoretic pattern of multiplex RT‐PCR products for the four positive patients for constitutional methylation and five normal controls, where M is the molecular weight marker (100 bp) and *B2M* is beta‐2‐microglobulin. Electropherogram of fragment analysis of the multiplex RT‐PCR products, with the relative peak areas of a positive case for constitutional methylation (B) and control (C) being shown. In the case with constitutional methylation, a decreased *MLH1* expression relatively to the internal control is evident.

To assess global *MLH1* transcript levels, we measured its relative expression levels by semiquantitative multiplex RT‐PCR in PBL samples of patients with constitutional methylation (#3, #10, #27, #38) and controls. We amplified the *MLH1* transcript and the *B2M* as internal control. As shown by the electrophoretic profile of the multiplex RT‐PCR products (Fig. 3A), we observed a decrease in *MLH1* RNA expression in the cases positive for constitutional methylation compared to controls.

To confirm and measure the differences in the transcript levels observed in the electrophoresis of the multiplex RT‐PCR products from patients with constitutional methylation and controls, we performed fragment analysis. Controls presented an average of 99% of *MLH1* transcript expression relatively to *B2M*. Considering the four cases with constitutional methylation, an average decrease in *MLH1* transcript levels of 38%, 37%, 46%, and 41% (when compared to *B2M*) was seen for patients #3, #10, #27, and #38, respectively (Fig. 3B, and C).

### *MLH1* allelic expression

**Figure 4:**
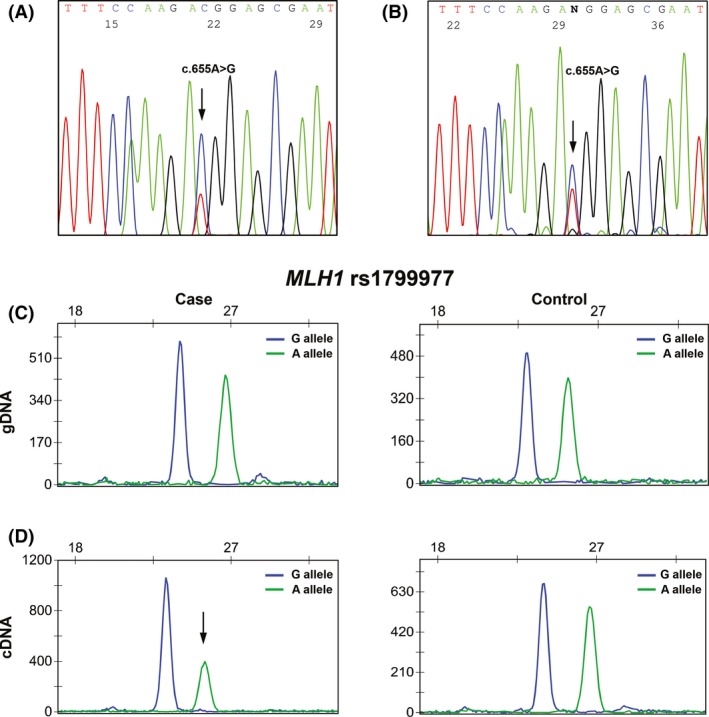
Electropherogram of sequencing analysis demonstrating the allelic expression of the SNP rs1799977 in exon 8 of *MLH1* in cDNA samples in Patient #10 (A) and in a control (B) (both reverse). *MLH1* rs1799977 (c.655A > G) SNuPE analysis in gDNA (C) and in cDNA (D) of an heterozygous patient with constitutional methylation (patient #27, left panels) and of a control (right panels). Partial transcriptional silencing of the A allele in the cDNA of the patient was observed.

Data available from the *MLH1* germline mutation screening showed that two of the probands positive for constitutional methylation of the *MLH1* promoter (patient #10 and #27) were heterozygous for the coding polymorphism c.655A>G, p.(Ile219Val) (rs1799977) in exon 8. The parents and sister of patient #27 were also studied for this polymorphism, the mother being homozygous for the A allele, and the father and sister heterozygous (Fig. 2C). In order to evaluate if the *MLH1* promoter methylation was monoallelic, the cDNAs of patients #10 and #27 were sequenced in PBL samples. In both cases, both alleles were present, but one of them appeared to be less expressed (Fig. 4A). This difference was more evident when compared with controls (Fig. 4B).

In order to confirm these results, we performed on gDNA and cDNA SNuPe analysis specific for the rs1799977 (c.655A>G), in the two constitutional methylation‐positive patients heterozygous for this polymorphism (cases #10 and #27) and in controls (also heterozygous). In the gDNA, no significant differences were obtained in the A/G allele relative areas (case #10: 86%; case #27: 86%; and controls: 88%, Fig. 4C). In the cDNA, we observed that both alleles were expressed in the two probands, but in both cases the A allele showed a signal reduction not observed in the controls (Fig. 4D), suggesting a decrease in the expression of the A allele. We observed a 45% decrease in the A allele relative to the G allele in patient #10 and a 27% decrease in patient #27, whereas in controls the difference between the two alleles was 2%. Moreover, the normalized ratios between cDNA and gDNA revealed a 53% and a 32% decrease in A allele in case #10 and in case #27, respectively, whereas in controls the normalized ratio difference was <0.1%.

## Discussion

In this study we identified four patients with constitutional methylation of *MLH1*. The frequency of constitutional *MLH1* methylation was 10.5% (4/38), signifying that *MLH1* methylation may account for a non‐negligible proportion of Lynch syndrome patients when analysis is restricted to those showing MLH1 abnormal expression in their tumors. In previous reports, the constitutional *MLH1* methylation frequency varied between 1.5 and 6% 9, 13, 19, 25, 27, 28, 29. Moreover, we show here that constitutional methylation appears to be specific of the *MLH1* gene in all four patients with epimutation. In fact, none of the other genes tested by MS‐MLPA and qMSP showed constitutional methylation, thus arguing against a generalized disruption of germline methylation patterns in these Lynch syndrome patients. Considering that we had identified 126 families with germline mutations in MMR genes in our institution at the time of this study, *MLH1* constitutional hypermethylation seems to be the molecular mechanism behind about 3% of Lynch syndrome families with molecular diagnosis at our institution.

Two of the four patients with constitutional *MLH1* methylation identified in this study had developed multiple Lynch syndrome‐associated tumors at an early age, one having metachronous (case #3) and the other synchronous tumors (case #38). This is in agreement with earlier reports of Lynch syndrome associated with constitutional methylation 8, 9, 10, 13, 19, 25. Regarding family history, three of four patients with constitutional *MLH1* methylation presented relatives with Lynch syndrome‐associated cancers, but their ages at diagnosis are compatible with sporadic origins. The fourth patient with constitutional *MLH1* methylation did not present family history of cancer, something that is quite common when this pathogenic mechanism is operative 8, 9, 10, 13, 19, 25.

Methylation of the *MLH1* promoter was studied in five regions, including the C‐ and D‐Deng regions. Methylation of the C‐Deng region has been directly correlated with transcriptional silencing and loss of MLH1 protein expression 18. The methylation level detected in PBL at the C‐Deng region by MS‐MLPA and qMSP were below 50% (Table 3), indicating that this alteration might be present in mosaic (the affected allele is not methylated in all cells), as described in other studies 10, 25, 28, 29. In these patients, methylation of the *MLH1* gene was also detected in other tissues, like normal colon mucosa (endoderm), oral mucosa (ectoderm), and muscle (mesoderm), representing all three germ layers. Thus, we can infer that in these cases the methylation of *MLH1* gene occurred early during embryogenesis 13, 19. Interestingly, we were able to study both the parents and sister of patient #27. None of them presented constitutional *MLH1* methylation, suggesting that in this patient *MLH1* methylation arose de novo, similar to most cases reported to date 9, 13.

The pattern of transmission of the different forms of epimutations in the *MLH1* gene depends on its origin and may be divided into primary and secondary. A dominantly transmitted constitutional *MLH1* methylation has been linked to a *MLH1* haplotype bearing two single‐nucleotide variants: c.‐27C>A and c.85G>T (p.Ala29Ser) 25, 30, 31. Studies of the c.−27C>A variant offer the most compelling evidence that *MLH1* promoter variants can directly affect the regulation of *MLH1* 9, 14, 18, 31. This variant has been associated with reduced transcriptional activity and the dominant inheritance of a mosaic constitutional *MLH1* methylation 11, 14. In our series this variant linked to secondary *MLH1* epimutation was not found. Sanger sequencing of the whole *MLH1* promoter (from c.−1469 to intron 1) was performed in PBL from all probands (*n *= 38) and in the available relatives (n = 3). In one case with *MLH1* promoter methylation (#38) the c.−269C>G variant was found in heterozygosity, but the association between these events is not known. No variants (besides common polymorphisms) in the *MLH1* promoter were found in the remaining three cases with *MLH1* promoter methylation, making it more likely that these patients have primary constitutional methylation, although we cannot exclude the remote possibility that there is a mutation in *cis* outside the regions studied causing secondary methylation.

When we compared the *MLH1* RNA expression in PBL samples (Table 3), we observed that there was significant loss of *MLH1* expression in the four cases with constitutional methylation of the *MLH1* gene when compared to controls. Our results are in agreement with other studies showing that constitutional methylation causes transcriptional downregulation of the *MLH1* gene 19, 25. On the other hand, we took advantage of an heterozygous polymorphism within *MLH1* exon 8 (rs1799977) in two patients to determine the effect of the methylation on *MLH1* transcriptional activity. In both cases, both alleles were present, but one of them was less expressed when compared with controls. The correlation between *MLH1* methylation and expression levels may not be directly proportional, and difficult to measure, given that different approaches, with different sensitivities, were used. However, our RNA studies have demonstrated a decrease in *MLH1* gene expression in cases with constitutional methylation, indicating that these events are associated. Recent studies have also shown that *MLH1* methylation can present itself as a constitutional alteration that results in the silencing of the affected allele 7, 25, 28, 31, 32, 33, 34, 35. Moreover, regarding the information about this polymorphism in patient #27 (heterozygous) and in the parents (father heterozygous and mother homozygous to the A allele), and considering the partial transcriptional silencing of the A allele observed in the cDNA of the proband, we can infer that the methylated allele is inherited from the mother (Fig. 2C). Interestingly, in the majority of sporadic cases reported in the literature, in whom the epimutations has arisen de novo, *MLH1* epimutation tended to occur on the maternal allele 7, 8, 28, 29, 34.

In patients with Lynch syndrome, the somatic inactivation, or “second hit”, of the wild‐type allele of the affected MMR gene leads to abnormal mismatch repair. This results in the accumulation of errors during DNA replication, especially in repetitive sequences known as microsatellites 34, 36. Consequently, tumors from patients with Lynch syndrome characteristically demonstrate MMR deficiency, defined as the presence of high microsatellite instability (MSI‐H) and/or the loss of MMR protein expression, which are the hallmarks of this disorder 37, 38. This feature is present in more than 90% of Lynch syndrome‐associated colorectal tumors in general and also in those associated with somatic or constitutional epigenetic silencing of the *MLH1* gene 19, 39, 40, 41. In fact, we found MSI‐H and loss of MLH1 expression, both at the mRNA and protein level, in all four tumors analyzed from the patients with *MLH1* constitutional methylation.

The p.Val600Glu *BRAF* mutation was absent in the tumors of the all four carriers of constitutional *MLH1* methylation reported here, as is the case for the majority of the cases in the literature 25, 28, 34, 40, 42. However, occasional reports 16, 28, 43 of *BRAF* mutation in CRC from patients with *MLH1* constitutional methylation exist. Until this issue is clarified in larger series, one should not exclude a patient with an early‐onset CRC from *MLH1* constitutional methylation testing based on the detection of a *BRAF* mutation in the tumor, as is currently common practice when selecting patients for *MLH1* germline mutation analysis.

We conclude that a significant proportion of patients with Bethesda criteria who have loss of MLH1 protein expression in their tumors and do not have a *MLH1* pathogenic germline mutation, display constitutional *MLH1* methylation as the mechanism of Lynch syndrome, especially in patients with CRC diagnosed before the age of 50 years, with multiple Lynch syndrome‐associated tumors, and no significant family history of early‐onset disease. Although the absolute risk of developing Lynch syndrome cancers in patients with *MLH1* constitutional methylation and the screening recommendations for relatives are not yet well defined, the inclusion of this analysis in the diagnostic strategy increases the diagnostic yield and allows screening and/or prophylactic measures in more patients with this syndrome. We therefore recommend constitutional *MLH1* promoter methylation analysis in all patients diagnosed <50 years of age with tumors presenting MLH1 loss of expression 44.

## Conflict of Interest

The authors declare that they have no competing interests.

## Supporting information


**Table S1.** Results of *MLH1* methylation using MS‐MLPA in PBL samples from all probands and controls.
**Table S2.** Results of *MLH1* methylation using MS‐MLPA CIMP kit in PBL samples from all probands.
**Table S3.** Results of *MLH1* methylation in a panel of genes by qMSP in PBL samples from all probands.
