# Supplementary material for: Contribution of MLH1 constitutional methylation for Lynch syndrome diagnosis in patients with tumor MLH1 downregulation
Source: Cancer Med. 2018 Jan 17;7(2):433–44. doi: 10.1002/cam4.1285 (PMC6193414; doi:10.1002/cam4.1285)
Supplement: Supplementary file 1 — Table S1. Results of MLH1 methylation using MS‐MLPA in PBL samples from all probands and controls. Table S2. Results of MLH1 methylation using MS‐MLPA CIMP kit in PBL samples from all probands. Table S3. Results of MLH1 methylation in a panel of genes by qMSP in PBL samples from all probands. [file CAM4-7-433-s001.docx]

**Supplementary Table 1 - Results of *MLH1* methylation using MS-MLPA in PBL samples from all probands and controls.**

|  | **% *MLH1* Methylation** | | | | |
| --- | --- | --- | --- | --- | --- |
|  | **A Region** | **B Region** | **C Region** | **D Region** | **Intron 1** |
| **Patient** | **(-659 nt)** | **(-382 nt)** | **(-246 nt)** | **(-13 nt)** | **(+206 nt)** |
| #1 | 4.1 | 0.0 | 0.0 | 1.6 | 0.0 |
| #2 | 0.0 | 0.0 | 0.0 | 1.6 | 0.0 |
| **#3** | **42.5** | **41.3** | **14.1** | **43.3** | **37.6** |
| #4 | 1.2 | 0.0 | 0.0 | 0.7 | 0.0 |
| #5 | 0.0 | 0.0 | 0.0 | 0.0 | 0.0 |
| #6 | 0.0 | 0.6 | 0.0 | 1.1 | 0.0 |
| #7 | 1.5 | 0.0 | 0.0 | 0.0 | 0.0 |
| #8 | 1.5 | 0.0 | 0.9 | 1.0 | 1.7 |
| #9 | 0.0 | 0.0 | 0.0 | 0.0 | 0.0 |
| **#10** | **49.9** | **45.9** | **18.3** | **49.9** | **43.6** |
| #11 | 1.4 | 0.0 | 0.0 | 0.0 | 0.0 |
| #12 | 0.0 | 0.0 | 0.0 | 0.0 | 0.0 |
| #13 | 1.7 | 0.0 | 0.0 | 0.8 | 0.0 |
| #14 | 2.0 | 0.0 | 0.0 | 2.5 | 0.0 |
| #15 | 1.7 | 0.0 | 0.0 | 0.9 | 0.0 |
| #16 | 2.3 | 0.0 | 0.0 | 1.5 | 0.0 |
| #17 | 6.4 | 0.0 | 0.0 | 1.6 | 0.0 |
| #18 | 1.2 | 0.0 | 0.0 | 0.0 | 0.0 |
| #19 | 1.1 | 0.0 | 0.0 | 0.0 | 0.0 |
| #20 | 0.0 | 0.0 | 0.0 | 0.0 | 0.0 |
| #21 | 0.0 | 0.0 | 0.0 | 0.0 | 0.0 |
| #22 | 3.2 | 0.0 | 0.0 | 0.0 | 0.0 |
| #23 | 0.6 | 0.0 | 0.0 | 0.0 | 0.0 |
| #24 | 0.0 | 0.0 | 0.0 | 1.0 | 0.0 |
| #25 | 0.9 | 0.0 | 0.0 | 0.0 | 0.0 |
| #26 | 0.0 | 0.0 | 0.0 | 0.0 | 0.0 |
| **#27** | **50.3** | **49.2** | **32.6** | **51.4** | **48.3** |
| #28 | 0.0 | 0.0 | 0.0 | 2.2 | 0.0 |
| #29 | 0.0 | 0.0 | 0.0 | 1.8 | 0.0 |
| #30 | 0.0 | 0.0 | 0.0 | 2.5 | 0.0 |
| #31 | 3.5 | 0.0 | 0.0 | 2.5 | 1.3 |
| #32 | 0.8 | 0.0 | 0.0 | 0.9 | 0.0 |
| #33 | 0.0 | 1.4 | 0.0 | 2.1 | 0.0 |
| #34 | 0.0 | 0.0 | 0.0 | 0.9 | 0.0 |
| #35 | 0.7 | 0.0 | 0.0 | 2.0 | 0.0 |
| #36 | 1.0 | 0.0 | 0.0 | 0.8 | 0.0 |
| #37 | 0.7 | 0.5 | 0.0 | 2.2 | 1.1 |
| **#38** | **51.0** | **54.8** | **46.3** | **52.3** | **51.3** |
| Controls | 0.9 | 0.7 | 0.0 | 1.7 | 0.0 |

**PBL**, peripheral blood lymphocytes.

The four probands positive for constitutional *MLH1* methylation are highlighted in bold.

**Supplementary Table 2 - Results of *MLH1* methylation using MS-MLPA CIMP kit in PBL samples from all probands**

|  | % Methylation by MS-MLPA (gDNA) | | | | | | | |
| --- | --- | --- | --- | --- | --- | --- | --- | --- |
| Patient | *CACNA1G* | *CDKN2A* | *CRABP1* | *IGF2* | ***MLH1*** | *NEUROG1* | *RUNX3* | *SOCS1* |
| #3 | 1.9 | 1.3 | 5.6 | 8.0 | **41.4** | 9.3 | 0.0 | 0.8 |
| #10 | 1.1 | 1.0 | 3.5 | 5.2 | **40.0** | 7.8 | 0.0 | 0.5 |
| #27 | 0.9 | 1.2 | 7.5 | 5.2 | **37.0** | 10.0 | 0.0 | 0.8 |
| #38 | 1.9 | 2.0 | 5.9 | 9.3 | **48.0** | 9.8 | 0.0 | 0.7 |
| Controls | 0.1 | 1.5 | 3.9 | 4.8 | **1.0** | 6.7 | 0.0 | 0.5 |

*MLH1* methylation levels are highlighted in bold.

**Supplementary Table 3 - Results of *MLH1* methylation in a panel of genes by qMSP in PBL samples from all probands**

|  | % Methylation by qMSP (gDNA) | | | | | | |
| --- | --- | --- | --- | --- | --- | --- | --- |
| Patient | *CNRIP1* | *FBN1* | *INA* | *MAL* | ***MLH1*** | *SNCA* | *SPG20* |
| #3 | 0.09 | 0.00 | 0.00 | 0.28 | **35.35** | 0.96 | 0.96 |
| #10 | 0.00 | 0.00 | 0.00 | 0.00 | **37.04** | 0.00 | 0.57 |
| #27 | 0.00 | 0.00 | 0.00 | 0.00 | **38.19** | 0.00 | 0.00 |
| #38 | 0.00 | 0.00 | 0.00 | 0.00 | **41.93** | 0.00 | 0.00 |

*MLH1* methylation levels are highlighted in bold.
